# Loss of Cochlear Ribbon Synapse Is a Critical Contributor to Chronic Salicylate Sodium Treatment-Induced Tinnitus without Change Hearing Threshold

**DOI:** 10.1155/2020/3949161

**Published:** 2020-07-25

**Authors:** Wei Zhang, Zhe Peng, ShuKui Yu, Qing-Ling Song, Teng-Fei Qu, Lu He, Ke Liu, Shu-Sheng Gong

**Affiliations:** ^1^Department of Otolaryngology-Head and Neck Surgery, Beijing Friendship Hospital, Capital Medical University, Beijing City Xi Cheng District Road 95 Yong an, Beijing 100050, China; ^2^Department of Otolaryngology-Head and Neck Surgery, Eye Hospital China Academy of Chinese Medical Science, Beijing City Shi Jingshan District Lugu Road 33, Beijing 100040, China

## Abstract

Tinnitus is a common auditory disease worldwide; it is estimated that more than 10% of all individuals experience this hearing disorder during their lifetime. Tinnitus is sometimes accompanied by hearing loss. However, hearing loss is not acquired in some other tinnitus generations. In this study, we injected adult rats with salicylate sodium (SS) (200 mg/kg/day for 10 days) and found no significant hearing threshold changes at 2, 4, 8, 12, 14, 16, 20, or 24 kHz (all *p* > 0.05). Tinnitus was confirmed in the treated rats via Behaviour Testing of Acoustic Startle Response (ASR) and Gap Prepulse Inhibition Test of Acoustic Startle Reflex (GPIAS). A immunostaining study showed that there is significant loss of anti-CtBP2 puncta (a marker of cochlear inner hair cell (HC) ribbon synapses) in treated animals in apical, middle, and basal turns (all *p* < 0.05). The ABR wave I amplitudes were significantly reduced at 4, 8, 12, 14, 16, and 20 kHz (all *p* < 0.05). No significant losses of outer HCs, inner HCs, or HC cilia were observed (all *p* > 0.05). Thus, our study suggests that loss of cochlear inner HC ribbon synapse after SS exposure is a contributor to the development of tinnitus without changing hearing threshold.

## 1. Introduction

Tinnitus is becoming a serious health problem worldwide [1–3]. It was proposed that tinnitus is probably induced by an imbalance between neuronal excitability and inhibition in the auditory circuit [4, 5]. The occurrence of tinnitus is associated with hearing loss, cochlear damage, and multiple types of the stress [6]. However, patients with normal audiogram may also exhibit tinnitus [7–10]. Clinical studies provided evidence that patients with both tinnitus and normal audiograms exhibit significant reduction in wave I amplitudes of auditory brainstem response (ABR) [11–13]. An alternative animal study showed that the mice exposed to noise develop temporary threshold shift (TTS) [14] and irreversible loss of cochlear ribbon synapses that connect cochlear inner HCs and spiral ganglion cells (SGCs) [13, 15]. However, it is unclear whether loss of cochlear ribbon synapses contributes to tinnitus generation in patients with normal audiogram. Bauer et al. found behavioural evidence indicating that loss of auditory nerve (AN) fibers in rats may associate with tinnitus [16]; however, there is significant loss of hearing and HCs in rats after noise exposure. Thus, this model may be unsuitable for exploring the etiology of tinnitus with normal audiogram. It is necessary to find an appropriate model to identify the correlations between tinnitus with normal audiogram and loss of cochlear ribbon synapses.

SS, an active component of the nonsteroidal anti-inflammatory drug aspirin, has been commonly used to generate tinnitus in animals [17]. SS is an anti-inflammation drug used to manage rheumatoid arthritis at therapeutic dose; SS inhibits cyclooxygenase activity and prostaglandin synthesis. High-dose SS could induce tinnitus characterized by TTS [11, 18]. However, it remains unknown whether such tinnitus features loss of cochlear ribbon synapses and/or cochlear HCs. Notably, the losses responsible for tinnitus accompanied by normal audiogram have not yet been identified.

Here, we present the evidence that an appropriate dose of SS exposure can cause tinnitus with normal audiogram and cochlear HCs, but loss of cochlear ribbon synapse suggesting loss of cochlear inner HC ribbon synapse may largely contribute to SS-induced tinnitus.

## 2. Materials and Methods

### 2.1. Animal

All studies were approved according to the Institutional Animal Care and Use Committee at the Capital Medical University of China. Wistar rats (adult, male, weighted 250 g~280 g) were obtained from the animal experimental ministry of Capital Medical University. Animals were divided into two groups according to intraperitoneal injection contents for ten days: (i) control group with injection of saline and (ii) SS-treated group with an injection of 5% (200 mg/kg) SS.

### 2.2. Behaviour Testing of ASR and GPIAS

ASR and GPIAS were used to measure tinnitus generation. Animals were placed in a permeable sound box resting on a sensitive piezoelectric capable of generating a voltage proportional to the magnitude of the startle responses, evoked by sound stimuli generated by a digital signal processor. The test apparatus was located in a soundproof chamber equipped with a tweeter on the chamber's ceiling about 10 cm above the rat's head. Animals were placed in the box for ten minutes prior to testing for adaptation. Test sessions include gap and no gap trial pair-arranged. The background sound is a pure tone of 12 k-16 k Hz, 70 dB, and the startle stimulus is 120 dB broad-banded noise (20 ms) in each trial. The gap lasting 75 ms was embedded in the background tone 100 ms prior to the startle stimulus. The maximum startle reflex within 250 ms after startle stimulus was recorded. Intertrial time was settled 12 sec to 21 sec randomly. ASR amplitude was recorded as the voltage generated by startle reflex of the tested animals (millivolt, mV). GPIAS was calculated as a ratio using the formula: 1 − (gap/no gap). Animals with tinnitus have been speculated to demonstrate poorer gap detection ability, which can be measured as a lack of suppression of the startle response in gap trial. Tinnitus rats present a lower GPIAS ratio. We compared the ASR amplitude and GPIAS inhibition rates of the SS-treated and control groups.

### 2.3. ABR Recordings

The evoked response signal-processing System 3 hardware (Tucker Davis Technologies, Alachua, FL, USA) and SigGen/BioSig software (Tucker Davis Technologies) were used for ABR measurement. Animals were anesthetized with Xylazine (10 mg/kg) and Ketamine (90 mg/kg) and placed in a soundproof chamber. Three needle electrodes were inserted at vertex (active) and beneath of each pinna (reference and ground), subcutaneously. The ABR threshold is the lowest stimulus intensity that produced reliable and reproducible ABR waves. We evaluated the rats hearing status before and at the end of this study and recorded ABR threshold at pure tone frequency of 2, 4, 8, 12, 16, 20, and 24 k Hz. Wave I amplitude was measured peak to baseline, and latency was measured within a specified time window at 90 dB level at each frequency.

### 2.4. Immunostaining

Animals were decapitated after anaesthesia, and the temporal bones were removed and fixed for 2 h in 10% formaldehyde in phosphate-buffered saline (PBS). After the samples were rinsed in PBS, the basilar membranes of the cochlea were dissected out for immunostaining. Permeabilized with 0.3% TritonX-100 (Sigma-Aldrich) for 30 min and blocked with 10% normal goat serum (Jackson) for 1 h; the tissues were incubated overnight at 4°C with the following primary antibodies: rabbit anti-myosin 7a (1 : 300, Proteus Biosciences); phalloidin 594 (1 : 500, Thermo Fisher); mouse anti-CtBP2 (1 : 300, BD); and chicken anti-NF200 (1 : 200,CHEMICOM). After rinsing with PBS, the samples were incubated in fluorescently labelled secondary antibodies (Alexa Fluor 488 and 568, Invitrogen/Molecular/Thermo Fisher) for 1 h at room temperature. Nuclei were visualized with 4′,6-diamidino-2-phenylindole (DAPI) (AppliChem).

### 2.5. Confocal Microscopy Imaging

Laser scanning confocal microscopy was conducted with a 63 X oil immersion objective lens (LEICA TCS SP8). Excitation wave lengths were 488, 568, and 594 nm, and local images were digitally magnified by twofold. Sequence scanning was performed from the apex and obtained at an interval of 0.5 *μ*m. Cross- sectioned cochleae were imaged covering the entire inner HC nucleus and areas beyond it in an image stack along the *z*-axis (*z*-stack).

### 2.6. Counting the Number of Ribbon Synapses, Outer HCs, and Inner HCs

Quantification of ribbon synapses, outer HCs, and inner HCs was performed in the cochlear apex turn, middle turn, and basal turn. Quantification was performed from the top of the cochlea to the bottom. In each basilar turn, we choose three visual fields containing approximately 9-11 inner HCs and 27-33 outer HCs. We selected five samples in each group to calculate the average number of ribbon synapses and HCs. The images were identified inner HC ribbon synapses by red fluorescence spot (indicating a pre synaptic ribbon) that appeared in each image. The total number of red fluorescence marks was obtained using Photoshop software. The number of marks in single inner HC was calculated. In the same way, the numbers of outer HCs and inner HCs in single image were calculated (green fluorescence staining).

### 2.7. Statistical Analysis

All data are presented as the mean ± SE. Statistical analysis was performed using GraphPad Prism 7 software (GraphPad Software Inc., La Jolla, CA, USA). Statistical differences between groups in ABR threshold shifts, HC counts, and synaptic counts were analyzed using one-way analysis of variance (ANOVA), followed by Bonferroni's multiple comparison test. *p* values < 0.05 were considered statistically significant.

## 3. Results

### 3.1. Behaviour Examination of Tinnitus Induced by SS

**Figure 1:**
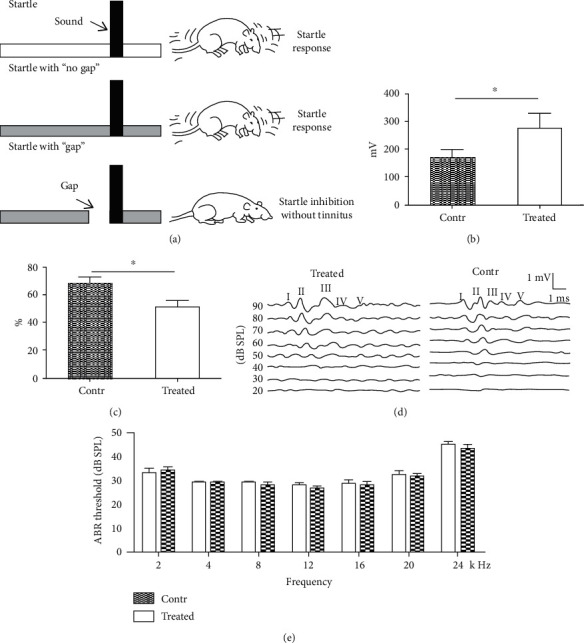
ASR and GPIAS procedures used to measure tinnitus and hearing threshold shifts before and after SS exposure. (a) Sketch map of behavior detection of tinnitus. The rat is startled in the presence of a sudden noise burst (startle stimulus). In normal animals, a silent gap in pure tone background prior to delivery of startle stimulus inhibits the startle response. The rats with putative tinnitus (sound condition is similar to the background tone) exhibit defective behavior. They do not identify the silent gap because of tinnitus. Thus, the startle response is not inhibited in rats with tinnitus, compared to controls without tinnitus. (b) ASR amplitudes of the control (dashed columns, *n* = 7) and SS-treated (blank columns, *n* = 7) groups. Average acoustic startle reflex amplitude was significant higher in the treated group than in the control group (^∗^*p* < 0.05). (c) GPIAS values of the control (dashed columns, *n* = 10) and SS-treated (blank columns, *n* = 11) groups; between-group differences were significant (^∗^*p* < 0.05). (d) Representative ABR waveforms evoked by pure tone in the SS-treated (upper left) and control (upper right) rats; acoustic intensities are graded from high (90 dB SPL) to low (20 dB SPL). Waveforms are labeled by I, II, III, IV, and V. (e) Statistical analysis of hearing threshold shifts between the SS and control groups across all frequencies tested (2, 4, 8, 12, 16, 20, and 24 kHz); no significant between-group differences were found at any frequency (all *p* > 0.05). *n* = 8 for the treated group and *n* = 6 for the control group.

Rats were intraperitoneally injected with SS (200 mg/kg/day) for 10 days. Tinnitus was evaluated via behaviour detections (Figure 1(a)). First, the ASR was evaluated in both groups. The mean amplitudes of startle reflex in the treated and control group animals were 274.91 ± 53.36 and 170.08 ± 28.61 (millivolt) (Figure 1(b)). The amplitude was significantly higher in the treated group than in the control group (*p* < 0.05). Next, GPIAS was measured in both groups. The mean ratios in the treated and control groups were 0.5145 ± 0.045 and 0.68 ± 0.0466 (ratio) (Figure 1(c)). The mean ratio was significantly lower in the treated group than in the control group (*p* < 0.05), consistent with the previous studies. These results indicate that SS-induced tinnitus.

### 3.2. SS Treatment Did Not Cause Elevations of ABR Threshold

To examine whether SS exposure in this study induced hearing loss, ABR thresholds of both groups were measured. Pure tone ABR testing was performed at 2, 4, 8, 12, 16, 20, and 24 kHz 2 h after the last SS injection. For the SS-treated group, the respective results were 33.13 ± 2.10, 29.38 ± 0.62, 29.38 ± 0.62, 28.13 ± 0.91, 28.75 ± 1.56, 32.50 ± 1.33, and 45.00 ± 1.33 dB SPL; for the control group, the respective results were 34.17 ± 1.53, 29.17 ± 0.83, 28.33 ± 1.05, 26.67 ± 1.05, 28.33 ± 1.05, 31.67 ± 1.05, and 43.33 ± 1.66 dB SPL (at 2, 4, 8, 12, 16, 20, and 24 kHz). These findings were not significantly different (Figures 1(d) and 1(e), all *p* > 0.05), indicating that SS did not cause hearing loss.

### 3.3. SS Exposure Did Not Induce Cochlear HC Loss

**Figure 2:**
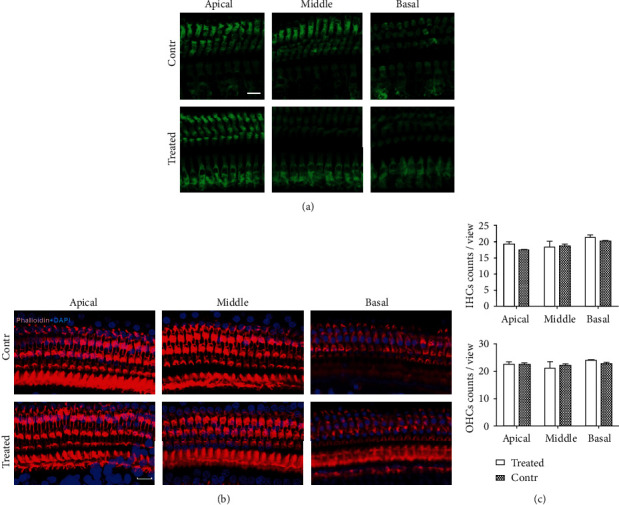
Cochlear outer and inner HC counts in the SS-treated and control rats. (a) Whole mounts of apical, middle, and basal turns were stained using myosin 7a (in green) to observe outer and inner HCs of the treated and control groups. Three rows of outer HCs and one row of inner HCs are evident; no obvious cell loss is apparent. Bar = 10 *μ*m. (b) Whole mounts of apical, middle, and basal turns were subjected to phalloidin staining (red) to trace HC hair bundles. No significant ciliary abnormality was apparent. Bar = 15 *μ*m. (c) Inner and outer HC counts of apical, middle, and basal turns of the SS-treated and control groups, respectively. Averages did not significantly differ between groups (all *p* > 0.05).

To explore whether SS exposure in the study caused the loss of cochlear HCs, we observed myosin 7a and phalloidin immunostaining to count the number of outer and inner HCs. This immunostaining revealed no differences in cell numbers among apical, middle, and basal turns in the SS group and control animals (Figures 2(a) and 2(c)); thus, SS did not induce the loss of cochlear HCs. No significant cilium loss was found from either outer or inner HCs after SS injection.

### 3.4. SS Exposure Caused Loss of Cochlear Inner HC Ribbon Synapses

**Figure 3:**
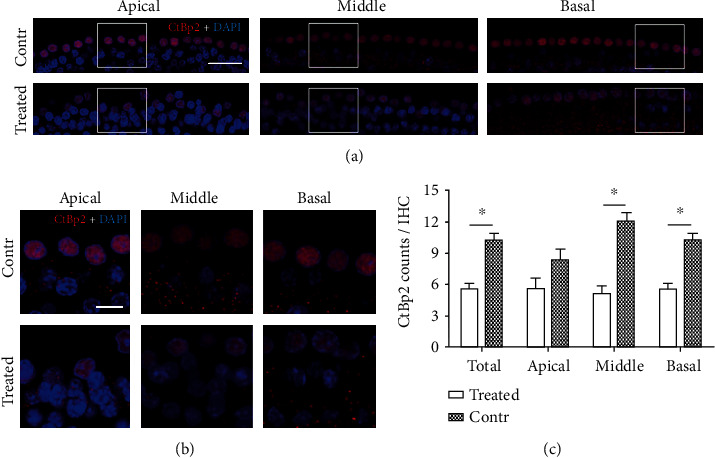
Quantitative analysis of cochlear ribbon synapses in the SS-treated and control groups. (a) Overview image of the whole mounts staining included apical, middle, and basal turns. Cochlear ribbon synapses were labeled using anti-CtBP2 staining (red, below inner HCs); cell nuclei were stained with DAPI (blue). CtBP2 was also present in the nucleus. Bar = 30 *μ*m. (b) Enlarged images of apical, middle, and basal turns in panels of (a) (white frames); CtBP2 staining puncta were counted in randomly chosen regions containing four inner HCs. SS significantly reduced CtBP2 puncta numbers. Bar = 10 *μ*m. (c) Quantitative analysis of cochlear ribbon synapses in the SS and control groups. The average number of CtBP2 staining puncta in apical, middle, and basal turns was analyzed, respectively. Total numbers of CtBP2 staining puncta in the two groups were also analyzed. There was a significant loss of CtBP2 staining puncta in SS-exposed rats compared to the control group (all *p* < 0.05).

To explore whether SS exposure triggered the loss of cochlear ribbon synapse, we applied RIBEYE/CtBP2 staining (specific for preribbon synapses). This staining was dramatically reduced in all three turns in the SS group (Figures 3(a) and 3(b)). The reductions of cochlear inner HCs ribbon synapses were statistically significant (Figure 3(c)).

### 3.5. SS Exposure Disrupted Cochlear Ribbon Synapse Function

**Figure 4:**
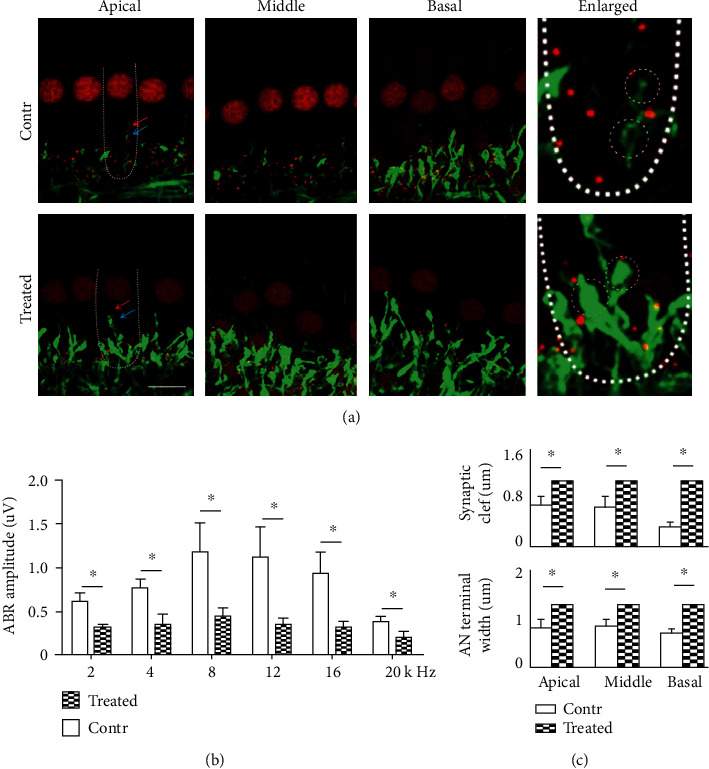
SS exposure causes swelling of fiber endings of postsynaptic ANs and amplitude changes in ABR wave I. (a) AN fibers were identified using anti-NF200 (green), and cochlear ribbon synapses were identified using anti-CtBP2 (red, blow inner HCs). Massively swollen AN fiber ends with reduced numbers of CtBP2-expressing puncta were evident in the SS group (compared to control group), as shown by white dashed lines (apical turns, left panel) and in enlargements (white circles, right panel). Bar = 10 *μ*m. (b). ABR wave I amplitudes at 2, 4, 8, 12, 16, and 20 kHz. Amplitudes of ABR wave I were found reduced across all the frequencies above (*p* > 0.05); there were significant differences across frequencies (*p* < 0.05); *n* = 8 in the treated group and *n* = 6 in the control group. (c) Cochlear ribbon synapse morphological properties. Synaptic clef was enlarged after SS treatment; there were significant differences in apical, middle, and basal turns (*p* < 0.05, top). Swelling at the AN terminals was found after SS treatment; there were significant differences in apical, middle, and basal turns (*p* < 0.05, bottom).

ABR wave I amplitudes were measured at 2, 4, 8, 12, 16, and 20 kHz; respective amplitudes were 462.6 ± 108.0, 433.0 ± 81.2, 320.8 ± 54.2, 362.7 ± 81.4, 217.3 ± 38.1, and 133.2 ± 17.2 nV in SS-treated animals, whereas they were 663.6 ± 90.5, 645.3 ± 90.18, 511.2 ± 71.3, 381.4 ± 48.3, 410.6 ± 54.0, and 254.2 ± 33.0 nV in control animals. These differences were statistically significant (Figure 4(b), all *p* < 0.05), indicating that ABR wave I amplitude decreased significantly in the rats after SS treatment. But no significant difference was found in ABR wave I latency between the two groups.

### 3.6. SS Exposure Affected Morphological Properties at or near the Cochlear Ribbon Synapses

This study explored whether SS exposure affected morphological properties at or near the synaptic connections between inner HCs and AN fibers. NF-200 staining (specific for nerve fibers) identified changes in auditory innervations. We found swelling fibers of ANs and overlapped puncta (as revealed by CtBP2 and NF200 costaining) after SS exposure (Figure 4(a)). SS induced abnormalities of both presynaptic elements and postsynaptic nerve fibers, thereby disrupting ribbon synapse function.

## 4. Discussions

HCs in the cochlea play a critical role in converting mechanical sound waves into neural signals for hearing [19–21]. Most hearing loss induced by noise, different ototoxic drugs, inflammation, or aging is caused by the HC damage [22–29]. Ribbon synapses are vital structures between inner HC and SGNs, which are the primary synaptic structures in the sound conduction pathway and play an important role in sound signal transmission [30–32]. Previous studies have shown that ribbon synapses are highly sensitive to noise in the cochlea [33–35]. Here, we found that SS-induced tinnitus did not cause the elevations of hearing threshold or cause loss of cochlear HCs. However, the cochlear ribbon synapses were lost, and the ends of afferent AN fibers near the sites of ribbon synapses were morphologically abnormal. Thus, SS-induced tinnitus with a normal audiogram may be associated with loss of cochlear ribbon synapses.

Kujawa and Liberman found that, after noise exposure, mice exhibited significant reductions in ABR wave I amplitudes, TTS, and loss of cochlear ribbon synapses [15]. However, they did not explore other auditory disorders such as tinnitus. Clinically, Schaette and McAlpine found that ABR wave I amplitudes were reduced in patients with tinnitus who exhibited normal audiograms [18]. But they did not explore damage to cochlear ribbon synapses. Here, it might be the first time that our study demonstrated the SS-induced tinnitus accompanied by a normal audiogram-featured loss of cochlear ribbon synapse.

Previous studies reported that different dosages of SS exposure can trigger tinnitus [17, 36]. SS-induced hearing threshold shifts are largely dose-dependent. For example, high-level SS exposure (>200 mg/kg) caused hearing impairment in both rats and mice [37, 38] and led to prolonged inner ear damage [39]. On the other hand, lower therapeutic doses of aspirin cause tinnitus but do not disrupt hearing threshold [40], consistent with our finding that a moderate SS dose (200 mg/kg/day) did not cause elevations of ABR thresholds.

Loss or damage of cochlear ribbon synapses will result in auditory disorder, regardless of whether hearing thresholds are affected. Cochlear ribbon synapses encode auditory information via fusion of massive neurotransmitter vesicles to achieve fast and tonic releasing [41, 42]. Synaptic dysfunction may underlie certain neuropsychiatric diseases, including Huntington's disease and autism spectrum disorders [41, 42]. Excessive glutamate release and consequent excitotoxic synaptic disruption contribute to the hearing loss after noise exposure [43]. TTS was found to have permanent loss of ribbon synapses, especially in the high-frequency cochlear region, accompanied by permanent reductions in ABR wave I amplitudes [44]. In this study, we found that ribbon synapse loss and ABR wave I amplitude reduction were both associated with tinnitus. Cochlear ribbon synapse loss is indicated by this type of wave reduction. However, there are other factors affecting ABR wave amplitudes besides cochlear ribbon synapse loss.

Noise exposure causes excessive neurotransmitter release and sodium influx into the postsynaptic terminal of the SGNs, leading SGN nerve (AN nerve) endings to swell. The resulting loss of synaptic connections is irreversible [45]. We found that SS treatment induced considerable loss of inner HC ribbon synapses, similar to that caused by noise exposure [45]. We also deduce that moderate SS exposure induced loss of both cochlear presynapses and postsynapses due to reported researches [31, 46, 47]. Further, we found that AN nerve endings were grossly swollen and no longer communicated with pre-synaptic structures, suggesting that SS exposure may also cause excitotoxicity to synaptic contacts between inner HCs and SGNs, triggering tinnitus. These findings are consistent with those of Zheng, who studied ototoxins that damaged auditory neurons and HCs. SS reduced the number of peripheral SGN neurons in vitro, but did not affect HC numbers. These findings suggest that the initial SS-induced hearing disorder may indicate neuronal dysfunction (e.g., involving afferent synapses) [48]. We infer that morphological changes evident at the ends of AN fibers may also contribute to reduction of ABR wave I amplitudes, which indicate lower cochlear outputs [49]. Tinnitus is presumed to involve both peripheral and central auditory systems. In this study, it may have been caused by damage to cochlear ribbon synapses and AN at an early stage of the peripheral auditory system.

Our study showed that moderate SS doses did not significantly change the numbers of outer or inner HCs, explaining the normal audiogram. It has been shown that moderate doses of SS do not affect auditory sensitivity, but steadily induce tinnitus [40, 50]. Here, we found that damage of cochlear ribbon synapse and AN triggered early-stage tinnitus, combined with a normal audiogram. In previous studies, SS treatment reduced the amplitudes of ABR wave I, consistent with functional loss of cochlear ribbon synapse [32, 50], although if cochlear ribbon synapse number is a time-dependent pattern during tinnitus induction or not was not observed. In summary, we found that loss of cochlear inner HCs ribbon synapses may play important role in the generation of tinnitus following moderate doses of SS. Notably, other factors may contribute. Future studies will focus on SGNs and other elements of auditory processing, as tinnitus is a complicated condition.

## References

[1] Baguley D., McFerran D., Hall D. (2013). Tinnitus. *The Lancet*.

[2] Ziai K., Moshtaghi O., Mahboubi H., Djalilian H. R. (2017). Tinnitus patients suffering from anxiety and depression: a review. *The International Tinnitus Journal*.

[3] Bauer C. A. (2018). Tinnitus. *The New England Journal of Medicine*.

[4] Lee A. C., Godfrey D. A. (2014). Cochlear damage affects neurotransmitter chemistry in the central auditory system. *Frontiers in Neurology*.

[5] Campbell J., Bean C., LaBrec A. (2018). Normal hearing young adults with mild tinnitus: reduced inhibition as measured through sensory gating. *Audiology Research*.

[6] Jay M., Bhatt H. W. L., Bhattacharyya N. (2016). Tinnitus epidemiology: prevalence, severity, exposures and treatment patterns in the United States. *JAMA Otolaryngology Head & Neck Surgery*.

[7] Barnea G., Attias J., Gold S., Shahar A. (1990). Tinnitus with normal hearing sensitivity: extended high-frequency audiometry and auditory-nerve brain-stem-evoked responses. *Audiology*.

[8] Qu T., Qi Y., Yu S. (2019). Dynamic changes of functional neuronal activities between the auditory pathway and limbic systems contribute to noise-induced tinnitus with a normal audiogram. *Neuroscience*.

[9] Guest H., Munro K. J., Plack C. J. (2017). Tinnitus with a normal audiogram: role of high-frequency sensitivity and reanalysis of brainstem-response measures to avoid audiometric over-matching. *Hearing Research*.

[10] Degeest S., Keppler H., Corthals P. (2017). The effect of tinnitus on listening effort in normal-hearing young adults: a preliminary study. *Journal of Speech, Language, and Hearing Research*.

[11] Gu J. W., Herrmann B. S., Levine R. A., Melcher J. R. (2012). Brainstem auditory evoked potentials suggest a role for the ventral cochlear nucleus in tinnitus. *Journal of the Association for Research in Otolaryngology*.

[12] Naomi F., Bramhall G. P. M., Gallun F. J., Konrad-Martin D. (2019). Auditory brainstem response demonstrates that reduced peripheral auditory input is associated with self-report of tinnitus. *The Journal of the Acoustical Society of America*.

[13] Bramhall N. F., Konrad-Martin D., McMillan G. P. (2018). Tinnitus and auditory perception after a history of noise exposure: relationship to auditory brainstem response measures. *Ear and Hearing*.

[14] Shi L., Liu K., Wang H. (2015). Noise induced reversible changes of cochlear ribbon synapses contribute to temporary hearing loss in mice. *Acta Oto-Laryngologica*.

[15] Kujawa S. G., Liberman M. C. (2009). Adding insult to injury: cochlear nerve degeneration after “temporary” noise-induced hearing loss. *The Journal of Neuroscience*.

[16] Bauer C. A., Brozoski T. J., Myers K. (2007). Primary afferent dendrite degeneration as a cause of tinnitus. *Journal of Neuroscience Research*.

[17] Guanyin Chen L. F., Liu Z., Sun Y., Chang H., Cui P. (2014). Both central and peripheral auditory systems are involved in salicylate-induced tinnitus in rats: a behavioral study. *PLoS One*.

[18] Schaette R., McAlpine D. (2011). Tinnitus with a normal audiogram: physiological evidence for hidden hearing loss and computational model. *The Journal of Neuroscience*.

[19] Yan Liu J. Q., Chen X., Tang M. (2019). Critical role of spectrin in hearing development and deafness. *Science Advances*.

[20] Qi J., Liu Y., Chu C. (2019). A cytoskeleton structure revealed by super-resolution fluorescence imaging in inner ear hair cells. *Cell Discovery*.

[21] Qi J., Zhang L., Tan F. (2020). Espin distribution as revealed by super-resolution microscopy of stereocilia. *American Journal of Translational Research*.

[22] He Z. H., Zou S. Y., Li M. (2020). The nuclear transcription factor FoxG1 affects the sensitivity of mimetic aging hair cells to inflammation by regulating autophagy pathways. *Redox Biology*.

[23] Gao S., Cheng C., Wang M. (2020). Blebbistatin inhibits neomycin-induced apoptosis in hair cell-like HEI-OC-1 cells and in cochlear hair cells. *Frontiers in Cellular Neuroscience*.

[24] Zhang Y., Li W., He Z. (2019). Pre-treatment with fasudil prevents neomycin-induced hair cell damage by reducing the accumulation of reactive oxygen species. *Frontiers in Molecular Neuroscience*.

[25] Liu W., Xu X., Fan Z. (2019). Wnt signaling activates TP53-induced glycolysis and apoptosis regulator and protects against cisplatin-induced spiral ganglion neuron damage in the mouse cochlea. *Antioxidants & Redox Signaling*.

[26] Zhang S., Zhang Y., Dong Y. (2020). Knockdown of Foxg1 in supporting cells increases the trans-differentiation of supporting cells into hair cells in the neonatal mouse cochlea. *Cellular and Molecular Life Sciences*.

[27] Tan F., Chu C., Qi J. (2019). AAV-ie enables safe and efficient gene transfer to inner ear cells. *Nature Communications*.

[28] He Z., Guo L., Shu Y. (2017). Autophagy protects auditory hair cells against neomycin-induced damage. *Autophagy*.

[29] He Z., Fang Q., Li H. (2019). The role of FOXG1 in the postnatal development and survival of mouse cochlear hair cells. *Neuropharmacology*.

[30] Matthews G., Fuchs P. (2010). The diverse roles of ribbon synapses in sensory neurotransmission. *Nature Reviews Neuroscience*.

[31] Coate T. M., Scott M. K., Gurjar M. (2019). Current concepts in cochlear ribbon synapse formation. *Synapse*.

[32] Goutman J. D. (2017). Mechanisms of synaptic depression at the hair cell ribbon synapse that support auditory nerve function. *Proceedings of the National Academy of Sciences of the United States of America*.

[33] Wang J., Yin S., Chen H., Shi L. (2019). Noise-induced cochlear synaptopathy and ribbon synapse regeneration: repair process and therapeutic target. *Advances in Experimental Medicine and Biology*.

[34] Luo Y., Qu T., Song Q. (2020). Repeated moderate sound exposure causes accumulated trauma to cochlear ribbon synapses in mice. *Neuroscience*.

[35] Fernandez K. A., Guo D., Micucci S., de Gruttola V., Liberman M. C., Kujawa S. G. (2020). Noise-induced cochlear synaptopathy with and without sensory cell loss. *Neuroscience*.

[36] Sheppard ShH A., Chen G.-d., Ralli M., Salvi R. (2014). Review of salicylate induced hearing loss neurotoxicity tinnitus and neuropathophysiology. *Acta Otorhinolaryngologica Italica*.

[37] Jiang C., Luo B., Manohar S., Chen G. D., Salvi R. (2017). Plastic changes along auditory pathway during salicylate-induced ototoxicity: hyperactivity and CF shifts. *Hearing Research*.

[38] Yu H., Vikhe Patil K., Han C. (2016). GLAST deficiency in mice exacerbates gap detection deficits in a model of salicylate-induced tinnitus. *Frontiers in Behavioral Neuroscience*.

[39] Chen G. D., Stolzberg D., Lobarinas E., Sun W., Ding D., Salvi R. (2013). Salicylate-induced cochlear impairments, cortical hyperactivity and re-tuning, and tinnitus. *Hearing Research*.

[40] Eggermont J. J. (2005). Tinnitus: neurobiological substrates. *Drug Discovery Today*.

[41] Toro R., Konyukh M., Delorme R. (2010). Key role for gene dosage and synaptic homeostasis in autism spectrum disorders. *Trends in Genetics*.

[42] Bourgeron T. (2009). A synaptic trek to autism. *Current Opinion in Neurobiology*.

[43] William R., Henry M. J. M. (1995). Afferent synaptic changes in auditory hair cells during noise-induced temporary threshold shift. *Hearing Research*.

[44] Rüttiger L., Singer W., Panford-Walsh R. (2013). The reduced cochlear output and the failure to adapt the central auditory response causes tinnitus in noise exposed rats. *PLoS One*.

[45] Wang D. Y., Wang Y. C., Weil D. (2010). Screening mutations of OTOFgene in Chinese patients with auditory neuropathy, including a familial case of temperature-sensitive auditory neuropathy. *BMC Medical Genetics*.

[46] Xiong W., Wei W., Qi Y. (2020). Autophagy is required for remodeling in postnatal developing ribbon synapses of cochlear inner hair cells. *Neuroscience*.

[47] Yang L., Chen D. S., Qu T. F. (2018). Maximal number of pre-synaptic ribbons are formed in cochlear region corresponding to middle frequency in mice. *Acta Oto-Laryngologica*.

[48] Zheng J. L., Gao W.-Q. (1996). Differential damage to auditory neurons and hair cells by ototoxins and neuroprotection by specific neurotrophins in rat cochlear organotypic cultures. *European Journal of Neuroscience*.

[49] Konadath S., Manjula P. (2016). Auditory brainstem response and late latency response in individuals with tinnitus having normal hearing. *Intractable & Rare Diseases Research*.

[50] Chen G.-D., Kermany M. H., D’Elia A. (2010). Too much of a good thing: long-term treatment with salicylate strengthens outer hair cell function but impairs auditory neural activity. *Hearing Research*.

